# Dysferlin Interacts with Tubulin and Microtubules in Mouse Skeletal Muscle

**DOI:** 10.1371/journal.pone.0010122

**Published:** 2010-04-12

**Authors:** Bilal A. Azakir, Sabrina Di Fulvio, Christian Therrien, Michael Sinnreich

**Affiliations:** 1 Neuromuscular Research Group, Montreal Neurological Institute and Hospital, McGill University, Montreal, Quebec, Canada; 2 Neuromuscular Center, Departments of Neurology and Biomedicine, University Hospital Basel, Basel, Switzerland; University of Birmingham, United Kingdom

## Abstract

Dysferlin is a type II transmembrane protein implicated in surface membrane repair in muscle. Mutations in dysferlin lead to limb girdle muscular dystrophy 2B, Miyoshi Myopathy and distal anterior compartment myopathy. Dysferlin's mode of action is not well understood and only a few protein binding partners have thus far been identified. Using affinity purification followed by liquid chromatography/mass spectrometry, we identified alpha-tubulin as a novel binding partner for dysferlin. The association between dysferlin and alpha-tubulin, as well as between dysferlin and microtubules, was confirmed in vitro by glutathione S-transferase pulldown and microtubule binding assays. These interactions were confirmed in vivo by co-immunoprecipitation. Confocal microscopy revealed that dysferlin and alpha-tubulin co-localized in the perinuclear region and in vesicular structures in myoblasts, and along thin longitudinal structures reminiscent of microtubules in myotubes. We mapped dysferlin's alpha-tubulin-binding region to its C2A and C2B domains. Modulation of calcium levels did not affect dysferlin binding to alpha-tubulin, suggesting that this interaction is calcium-independent. Our studies identified a new binding partner for dysferlin and suggest a role for microtubules in dysferlin trafficking to the sarcolemma.

## Introduction

Mutations in dysferlin cause limb girdle muscular dystrophy 2B (LGMD2B) [1], Miyoshi Myopathy (MM) [2] and distal anterior compartment myopathy [3]. Dysferlin is a large type II transmembrane protein composed of seven C2 domains and two Dysf domains [4]. The protein is predominantly expressed in skeletal and cardiac muscles and has also been reported to be expressed in the placenta [5]. Dysferlin is found in the sarcolemma and the t-tubular system of muscle fibres and was co-purified with the dihydropyridine receptor, a membrane protein present in the t-tubule structure [6]. Dysferlin was also shown to interact, via immunoprecipitation studies, with several cytosolic and membrane-associated proteins, such as MG53, affixin, annexins A1 and A2, AHNAK, caveolin-3 and calpain-3 [7], [8], [9], [10], [11], [12]. Dysferlin, annexin A1 as well as m-and mu-calpains, but not calpain-3, were shown independently to participate in membrane resealing, suggesting that these proteins could work synergistically to promote Ca^2+^-dependent membrane fusion and actin remodelling near the disruption site [13], [14], [15], [16]. Sarcolemmal repair is thought to occur by membrane patch formation through the fusion of subsarcolemmal vesicles located in proximity to the disruption site [13]. The source of these vesicles is still under debate but may implicate lysosome-derived vesicles and/or enlargeosomes, a new type of cytoplasmic vesicles that undergo rapid calcium-dependent, tetanus toxin insensitive exocytosis, and harbor as a luminal marker the dysferlin binding protein AHNAK [9], [17]. Caveolin-3 was shown to be implicated in the trafficking of dysferlin to the plasma membrane and to regulate the endocytosis of dysferlin [18].

We set out to identify additional dysferlin binding partners. Using affinity purification combined with liquid chromatography/mass spectrometry (LC-MS/MS), we identified alpha-tubulin as a novel binding partner for dysferlin in mouse skeletal muscle. Alpha- and beta-tubulin are the most common members of the tubulin family. Heterodimers composed of alpha- and beta-tubulin are needed for the polymerization of microtubules. Microtubules are dynamic structures that undergo continuous assembly/disassembly and are implicated in cellular motility, intracellular transport, mitosis and in the determination of cell morphology. During the differentiation of myoblasts, the microtubules are reorganised [19]. In myoblasts, microtubules nucleate at the centrosome and project towards the plasma membrane. In mature skeletal muscle cells, microtubules adopt longitudinal structures that run parallel to the sarcolemma [19], [20].

In this study, we sought to characterize the interaction between dysferlin and alpha-tubulin in muscle cells and tissues. The interaction detected by LC-MS/MS was further characterized by co-immunoprecipitation assays using recombinant or native proteins, as well as by direct binding assays with purified proteins and by confocal microscopy.

## Materials and Methods

### Ethics Statement

All animals were handled in strict accordance with good animal practice as defined by the relevant national and/or local animal welfare bodies, and all animal work was approved by the appropriate committee: Animal Care Committee and Institutional Review Board of the Montreal Neurological Institute, McGill University, Montreal, Canada.

### Cells, Animals, Plasmids and Antibodies

The human embryonic kidney-derived cell line, HEK293T, and the mouse myoblast-derived cell line, C2C12, were purchased from ATCC (Burlington, Ontario; ATCC number CRL-1573 and CRL-1772, respectively). CD1 mice were purchased from Charles River (Montreal, Canada).

The GFP-His-myc tagged dysferlin cDNA cloned into DSC-B plasmid was kindly provided by Dr K. Bushby (Newcastle, U.K) [21]. In this construct, the GFP coding sequence is located at the 5′ end, and the His-myc tags are located at the 3′ end of the dysferlin cDNA. For the experiments in which only His-myc-dysferlin was used, the GFP tag was removed by EcoRI/NotI restriction enzyme digestion.

The TubA4A and TubA1B constructs were generated from commercially available cDNA clones in pCMV6-XL5 vector (Origene). EcoRI and NotI restriction sites were included in the primer sequence to facilitate subcloning of the PCR fragment into pGEX4T1 (GE Healthcare) in order to generate GST fusion proteins.

The cloning of the GST recombinant dysferlin C2 domains was described previously [22]. All of the GST fusion proteins were expressed in *E.coli* BL21 and purified with glutathione-Sepharose 4B beads according to the manufacturer's instructions (Amersham Biosciences) and kept at 4°C until needed.

Monoclonal antibodies against c-myc, dysferlin, alpha-tubulin and GFP were purchased from the following suppliers, respectively: Biomol International, Vector laboratories, Invitrogen and Roche Diagnostics. For the detection of GST, we used goat polyclonal antibodies from GE Healthcare. Goat anti-rabbit IgG-HRP conjugate was purchased from Invitrogen and Cy™3-conjugated AffiniPure goat anti-mouse IgG antibody was purchased from Jackson Immunoresearch laboratories.

### Co-immunoprecipitation and Pulldown Assays

Transfected HEK293T cells grown in 100-mm dishes were washed with PBS and resuspended in 1 ml/dish with buffer A (10 mM HEPES, pH 7.4, protease inhibitors (Roche complete cocktail)). The cells were sonicated and Triton X-100 was added to a final concentration of 1%. Protein extracts were incubated for 20 min at 4°C and centrifuged at 13,000 rpm. For co-immunoprecipitation assays, extracts of transfected cells were precleared with protein A-Sepharose beads. The precleared supernatants were then incubated with the indicated antibodies and protein A-Sepharose beads for 16 h at 4°C. Beads were washed extensively with buffer A with 1% Triton X-100 and prepared for Western blot analysis by resuspension in 50 µl of buffer containing 2%SDS, 0.5% β-mercaptoethanol and 2% glycerol and heating to 95°C for 5 minutes prior to loading on a 3%–12% polyacrylamide gradient SDS-page gel.

For dysferlin C2 domain GST pulldown assays, C2 domain sequences were cloned into pGEX4T1 vectors, expressed in *E. coli* BL21 and purified by affinity chromatography with glutathione Sepharose 4B beads, as described in [22]. C2C12 myoblasts were lysed in a buffer containing 10 mM HEPES, pH 7.4, 33 mM NaCl and protease inhibitors (10 µM aprotinin, 10 µM leupeptin, 1 mM benzamidine, 1 mM PMSF, 1 µM E-64). After centrifugation at 13,000 rpm, 1 mg of C2C12 lysate was incubated overnight at 4°C with 10 µg of each GST-C2 domain fusion protein immobilized on glutathione Sepharose 4B beads, in the presence of 1 mM CaCl_2_ or 1 mM EGTA. The beads were washed extensively in the same buffer and prepared for Western blotting.

For the GST-TubA4A and GST-TubA1B pulldown assay and the calcium titration assay, dissected leg muscles from 4 week old mice were homogenized in a buffer containing 50 mM HEPES pH 7.6, 150 mM NaCl, 20 mM pyrophosphate sodium, 10 mM NaF, 2 mM sodium orthovanadate, 1% Triton X-100, 10% glycerol, 1 mM MgCl_2_ and protease inhibitor cocktail (Roche). After centrifugation at 100,000×*g*, 1 mg of protein was incubated overnight at 4°C with 10 µg of each GST-TubA fusion-protein isoform immobilized on glutathione Sepharose 4B beads. The beads were washed extensively in the same buffer and prepared for Western blotting. For the calcium titration assay, 1 mg of protein extracts were immunoprecipitated with protein A-Sepharose coupled to anti-alpha-tubulin antibody in presence of 1 mM of EGTA or increasing concentration of calcium. For this experiment, EDTA in the lysis buffer was compensated by adding a neutralizing amount of calcium, with subsequent calcium titration. The beads were washed extensively in the same buffer and prepared for Western blotting.

### Purification of His-myc-dysferlin

His-myc-dysferlin was overexpressed in HEK293T cells, captured on Ni-NTA beads using buffer A with 20 mM imidazole. Beads were washed in PBS buffer containing 50 mM imidazole, and His-myc-dysferlin was then eluted with 400 mM imidazole. The buffer was exchanged with 10 mM HEPES buffer pH 7.4 plus protease inhibitors using an Amicom Ultra centrifugal filter device (Millipore) in order to remove the imidazole, which is known to depolymerize microtubules [23].

### Western Blot

The membrane was blocked for 1 hour in buffer containing PBS-T (0.1% Tween-20) + 5% milk then incubated 16 h with the appropriate antibody (1∶1000) in the same buffer. The membrane was washed with the same buffer then incubated 1 hour with anti-mouse or anti-rabbit IgG HRP-conjugated antibody in the same buffer (1∶1000 dilution). The membrane was washed in PBS and detected by Amersham Hyperfilm ECL (GE Healthcare).

### Liquid Chromatography- Mass spectrometry (LC-MS/MS)

We used the service of the IRCM (Institut de recherches cliniques de Montréal) for the LC-MS/MS analysis. The method used for the LC-MS/MS analysis was described in detail in [24]. Briefly, the entire SDS-PAGE gel lane of co-purified dysferlin binding partners as well as its control lane were each excised into 14 bands and then cut into 1 mm^3^ pieces. Minced gel pieces were destained in 100 mM sodium thiosulfate and 30 mM potassium ferricyanide for 15 min, followed by a 5 min wash with 50 mM ammonium bicarbonate. Gel pieces were dehydrated with acetonitrile, reduced with a buffer containing 10 mM dithiothreitol and 100 mM ammonium bicarbonate for 30 min at 40°C, and then alkylated with a buffer containing 55 mM iodoacetamide and 100 mM ammonium bicarbonate for 20 min at 40°C. Gel pieces were dehydrated and washed at 40°C by adding acetonitrile for 5 min. Gel pieces were dried for 5 min at 40°C and then re-hydrated at 4°C for 40 min with the trypsin solution (6 ng/µl of sequence grade trypsin in 25 mM ammonium bicarbonate). Proteins were digested at 58°C for 1 h. Peptides were extracted with a buffer containing 1% formic acid and 50% acetonitrile. Peptide extracts were loaded on a LC column, which was a 75 µm ID fused silica capillary of 100 mm length packed with the Jupiter 5 µm C18 300 Å reverse-phase material (Phenomenex). This column was installed on the nanoLC-2D system (Eksigent) and coupled to the LTQ Orbitrap (ThermoFisher Scientific).

The mass resolution for MS was set to 30,000 (at m/z 400). Protein database searching was performed with Mascot 2.1 (Matrix Science) against the human NCBInr protein database.

### Microtubule Binding Assay

His-myc-dysferlin was prepared as described above. The microtubule binding assay was performed according to the manufacturer's instructions (Cytoskeleton, microtubule binding protein spin-down assay kit, Cat.# BK029). Briefly, MTs were prepared from 100 µg of tubulin monomers in 20 µl of PEM containing 1 mM GTP and 5% glycerol at 35°C for 20 min and immediately stabilized in 200 µl of PEM-20 µM Taxol. The MTs (4 µM tubulin) were incubated with 3 µg of purified His-myc-dysferlin for 30 minutes at room temperature in a reaction volume of 50 µl. The reaction mixtures were then centrifuged at 100,000×*g* at 25°C for 15 min in the presence of a 50% glycerol cushion-PEM-Taxol gradient. Proteins in the supernatant and pellet were separated on a 5–16% gradient polyacrylamide gel and visualized by Coomassie blue staining. BSA was used as a negative control and the microtubule binding fraction (MAPF) provided by the cytoskeleton assay kit containing MAP2A, MAP2B, MAP1 and tau as a positive control, where MAP2 constitutes 70% of MAPF.

### Immunofluorescence

C2C12 myoblasts were seeded on poly-D-lysine-coated coverslips and transfected with GFP-dysferlin using Lipofectamine 2000 (Invitrogen) and incubated for 16 h. Myotube formation was induced by switching the cells to DMEM media plus 5% horse serum (Gibco) for 5 days [25]. Cells were fixed with 4% paraformaldehyde in PBS for 4 minutes, permeabilized with 0.2% Triton X-100 in PBS for 4 minutes, blocked with 0.4% BSA and stained with anti-tubulin monoclonal antibody for 1 hour and finally incubated with Cy™3-conjugated AffiniPure goat anti-mouse IgG antibody. Images were captured on Zeiss LSM 710 inverted confocal microscopy using the Zen 2008 image analysis system.

## Results

### Identification of alpha-tubulin as a novel dysferlin protein interactor

**Figure 1:**
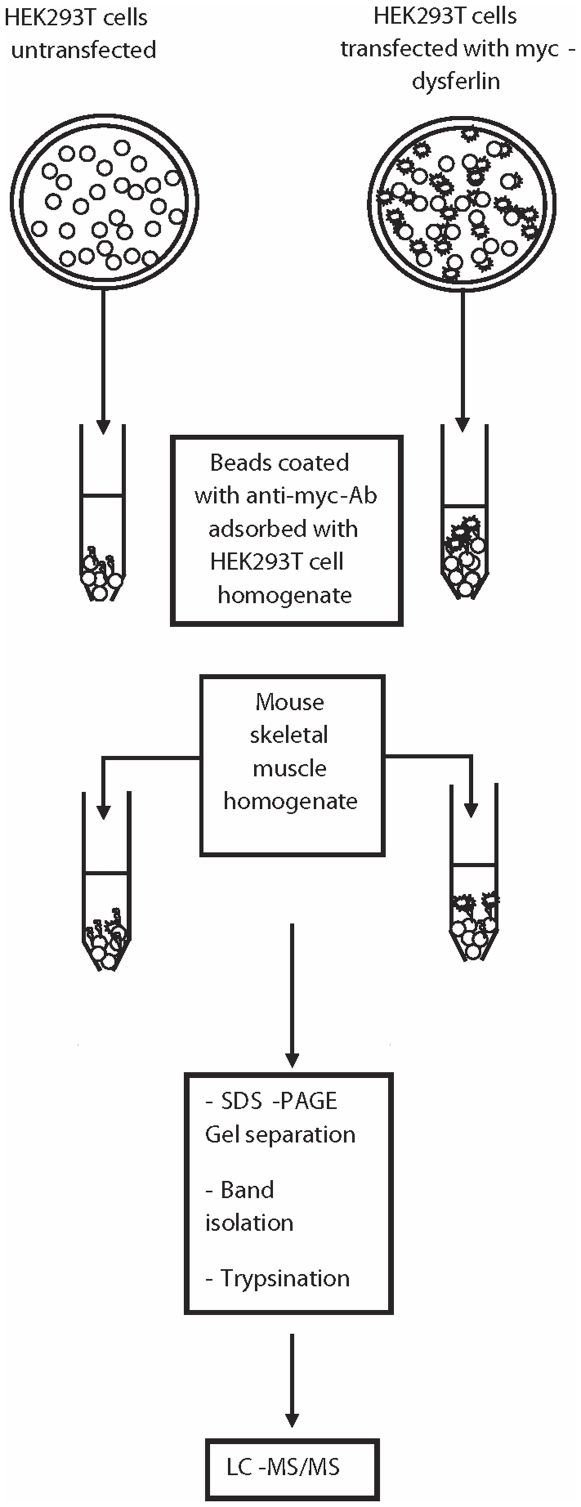
Schematic representation of the proteomic analysis by mass spectrometry. The procedure includes overexpression of His-myc-dysferlin in HEK293T cells, purification on protein A-Sepharose beads coupled to anti-myc antibody, incubation of the myc-dysferlin beads with mouse skeletal muscle homogenate and the identification of the co-purified proteins using LC-MS/MS.

**Figure 2:**
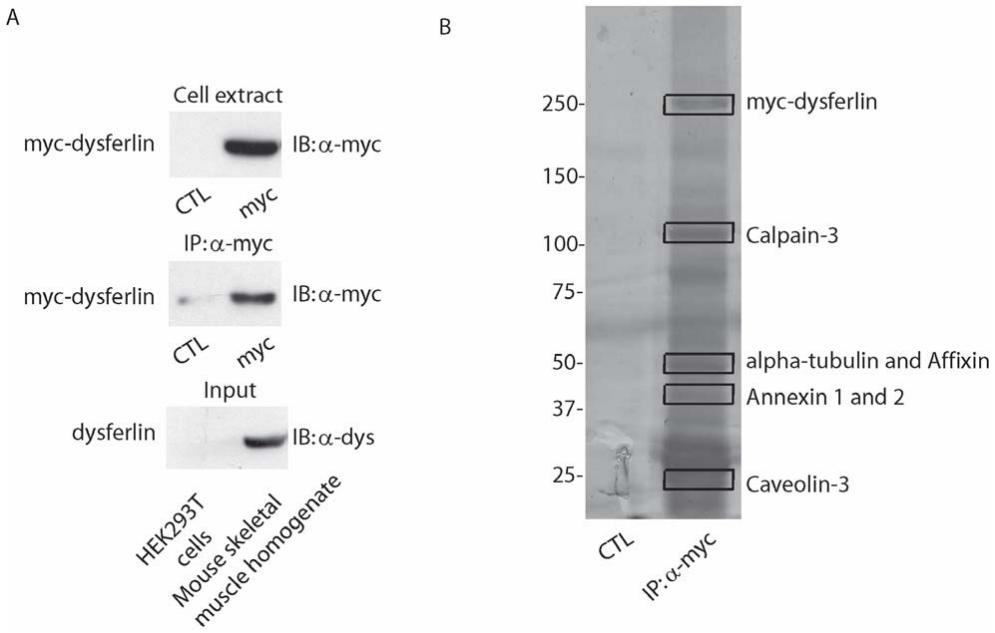
Alpha-tubulin is identified as a dysferlin interacting protein through affinity purification and mass spectrometry analysis. **A.** Western blot to detect the expression of His-myc-dysferlin in transfected HEK293T cells (top panel), the immunoprecipitated myc-dysferlin (middle panel) and expression of endogenous dysferlin in mouse skeletal muscle homogenate (bottom panel). **B.** SDS-PAGE resolution of the co-purified dysferlin partner stained with silver nitrate. Left lane is the control, whereby His-myc-dysferlin was not transfected into HEK293T cells. Right lane is the immunoprecipitate with myc-dysferlin. Bands containing alpha-tubulin and previously described dysferlin binding partners are highlighted. IB: Immunoblot, IP: Immunoprecipitation, CTL: control.

To identify dysferlin binding partners, we carried out Liquid chromatography-Mass spectrometry (LC-MS/MS) analysis on proteins obtained by pulldown with His-myc-dysferlin from mouse skeletal muscle homogenate (Figure 1). His-myc-dysferlin was overexpressed in HEK293T cells and purified by affinity from the cell lysates using protein A-Sepharose beads coated with anti-myc monoclonal antibodies. Beads were extensively washed and incubated with mouse skeletal muscle homogenate. Extract from non-transfected HEK293T cells was used as a negative control; since these cells do not express any detectable amounts of dysferlin (Fig. 2A, lower panel). After extensive washing, affinity-purified complexes were separated by SDS-PAGE and visualized by silver staining (Fig. 2B). The entire gel lane for both the control and the pulldown were each excised into 14 bands, and each band was cut into 1 mm^3^ pieces which underwent subsequent in-gel trypsin digestion. The resulting peptides were separated and analyzed with a nanoscale liquid chromatography quadrupole time of flight MS/MS automated system, according to the method described in [24]. Protein database searching was performed with Mascot 2.1 (Matrix Science) against the human NCBInr protein database. The experiment was repeated twice and proteins found in both control and pulldown were discarded as false positives.

We identified proteins originating from both HEK293T cells (identified by LC-MS/MS as being of human origin) as well as from mouse skeletal muscle (identified by LC-MS/MS as originating from mouse species) that showed significant association with dysferlin, and we focused on those proteins originating from mouse muscle. Importantly, we were able to identify the few proteins previously reported to interact with dysferlin, such as caveolin-3, affixin, AHNAK, annexin A1 and annexin A2 as well as calpain-3 (Fig. 2B), thus validating our approach as being able to detect dysferlin interacting partners. The newly identified proteins revealed in our analysis fall into several categories: surface membrane proteins, proteins involved in cellular trafficking, regulatory proteins and proteins related to the ubiquitin system. A 55 kDa band was found in the pulldown assay and contained 44 peptides matching to alpha-tubulin (Fig. 2B). Knowing that microtubules are used as trafficking pathway by the majority of the proteins that are targeted to the plasma membrane, we sought to study the interaction between dysferlin and alpha-tubulin.

### Alpha-tubulin binds to dysferlin in muscle cells

**Figure 3:**
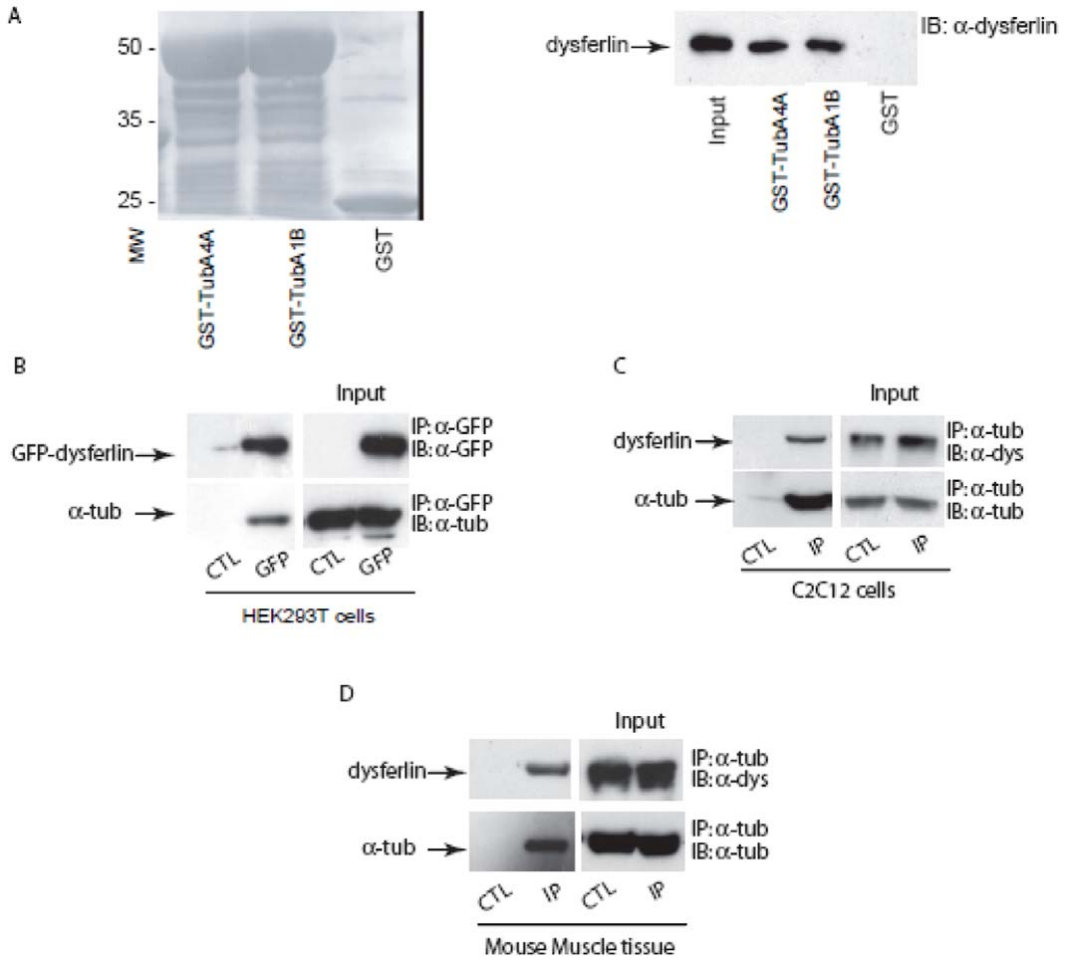
Dysferlin complexes with alpha-tubulin. **A.** GST, GST-TubA4A and GST-TubA1B fusion proteins immobilized onto glutathione-Sepharose 4B beads were incubated with mouse skeletal muscle homogenate. GST, GST-TubA4A or GST-TubA1B with adsorbed proteins from mouse skeletal muscle were resolved by SDS-PAGE, transferred onto a nitrocellulose membrane, and blotted with mouse monoclonal anti-dysferlin antibody. Left panel: nitrocellulose membrane stained with ponceau red, right panel: detection of immunoreactive dysferlin. **B.** GFP-dysferlin was overexpressed in HEK293T cells and then immunoprecipitated from cell extracts with anti-GFP antibody. As a control, protein A-Sepharose beads coated with anti-GFP antibody were incubated with extracts of non-transfected HEK293T cells. Proteins were separated on SDS-PAGE gel and were transferred onto a nitrocellulose membrane and blotted with anti-GFP or anti-alpha-tubulin antibodies. Input (right panel), immunoprecipitate (left panel). **C–D.** Co-immunoprecipitation of dysferlin with anti-alpha-tubulin antibody from C2C12 myotube extracts (**C**) or mouse skeletal muscle homogenate (**D**). Input (right panel), immunoprecipitate (left panel). As a control (CTL), protein A-Sepharose beads were incubated with myotube extracts in the absence of anti-alpha-tubulin antibody.

In order to confirm the interaction between alpha-tubulin and dysferlin found through the LC-MS/MS analysis, we undertook a series of protein binding and co-immunoprecipitation studies using recombinant and native proteins. Using immobilized recombinant GST-tubulin, we were able to detect binding interactions through the capture of endogenous dysferlin from mouse skeletal muscle (Fig. 3A). Interestingly, both isoforms of alpha-tubulin were able to capture murine dysferlin. TubA4A is expressed mainly in the testis and TubA1B is ubiquitous.

The interaction between dysferlin and alpha-tubulin in cells was addressed using a heterologous system. GFP-dysferlin was expressed in HEK293T cells and used for immunoprecipitation with anti-GFP antibodies. Native alpha-tubulin was found to co-immunoprecipitate with recombinant GFP-dysferlin (Fig. 3B). To account for any putative artefacts caused by an overexpression system, we next performed immunoprecipitation assays using C2C12 myotubes or mouse skeletal muscle homogenate with anti-alpha-tubulin antibodies. Native dysferlin co-immunoprecipitated with alpha-tubulin in muscle cells or tissues (Fig. 3C, Fig. 3D). A faint band is present in the control lane of Fig. 3C which we consider non-significant. Taken together, these results suggest that dysferlin interacts with alpha-tubulin *in vitro* as well as *in vivo*.

### Alpha-tubulin interacts with dysferlin's C2A and C2B domains in a calcium-independent manner

**Figure 4:**
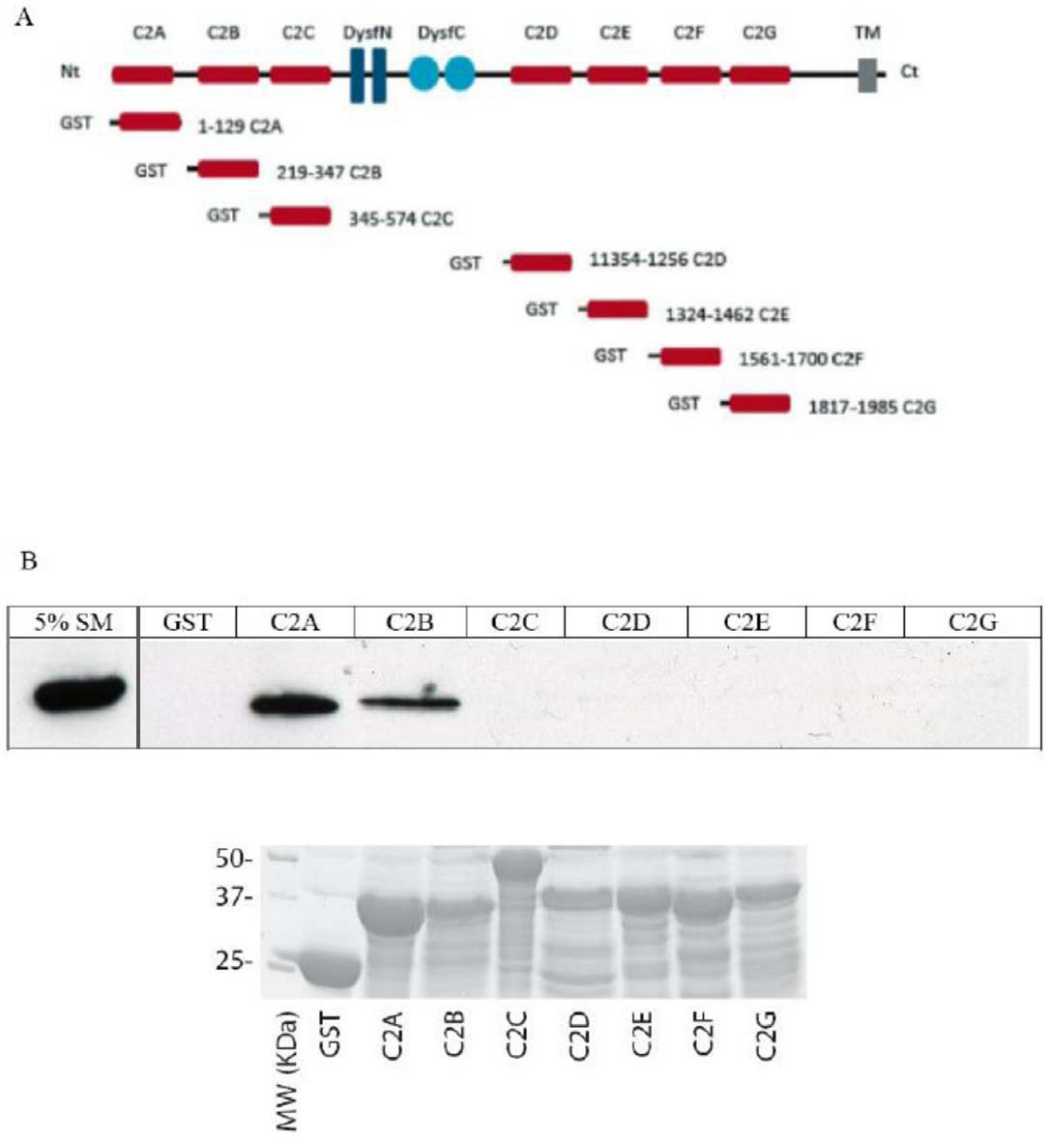
Alpha-tubulin interacts with dysferlin through the C2A and C2B domains. **A.** Schematic illustration of full-length wild type dysferlin and the various GST-C2 domain constructs used. **B.** C2C12 myoblast extract was incubated with GST alone or with the various GST-C2 domains precoupled to glutathione-Sepharose 4B beads. The bound proteins were separated on SDS-PAGE followed by Western blot analysis using anti-alpha-tubulin antibody. SM: standard material. Lower panel: nitrocellulose membrane of GST-dysferlin C2 domains with adsorbed proteins from the cell extract stained with ponceau red.

Since dysferlin is a multi-domain protein, we sought to identify which of dysferlin's seven C2 domains are involved in the interaction with alpha-tubulin (Fig. 4A). Recombinant GST-dysferlin C2 domain fusion proteins were immobilized on glutathione-Sepharose 4B beads and were incubated with C2C12 myoblast extract. Dysferlin-bound alpha-tubulin was detected by Western blotting with anti-alpha-tubulin antibodies. Alpha-tubulin was able to bind only to the C2A and C2B domains of dysferlin, while the other C2 domains did not bind to alpha-tubulin (Fig. 4B). The absence of alpha-tubulin binding to the GST moiety alone indicated that the binding to the dysferlin C2 domains A and B was specific.

**Figure 5:**
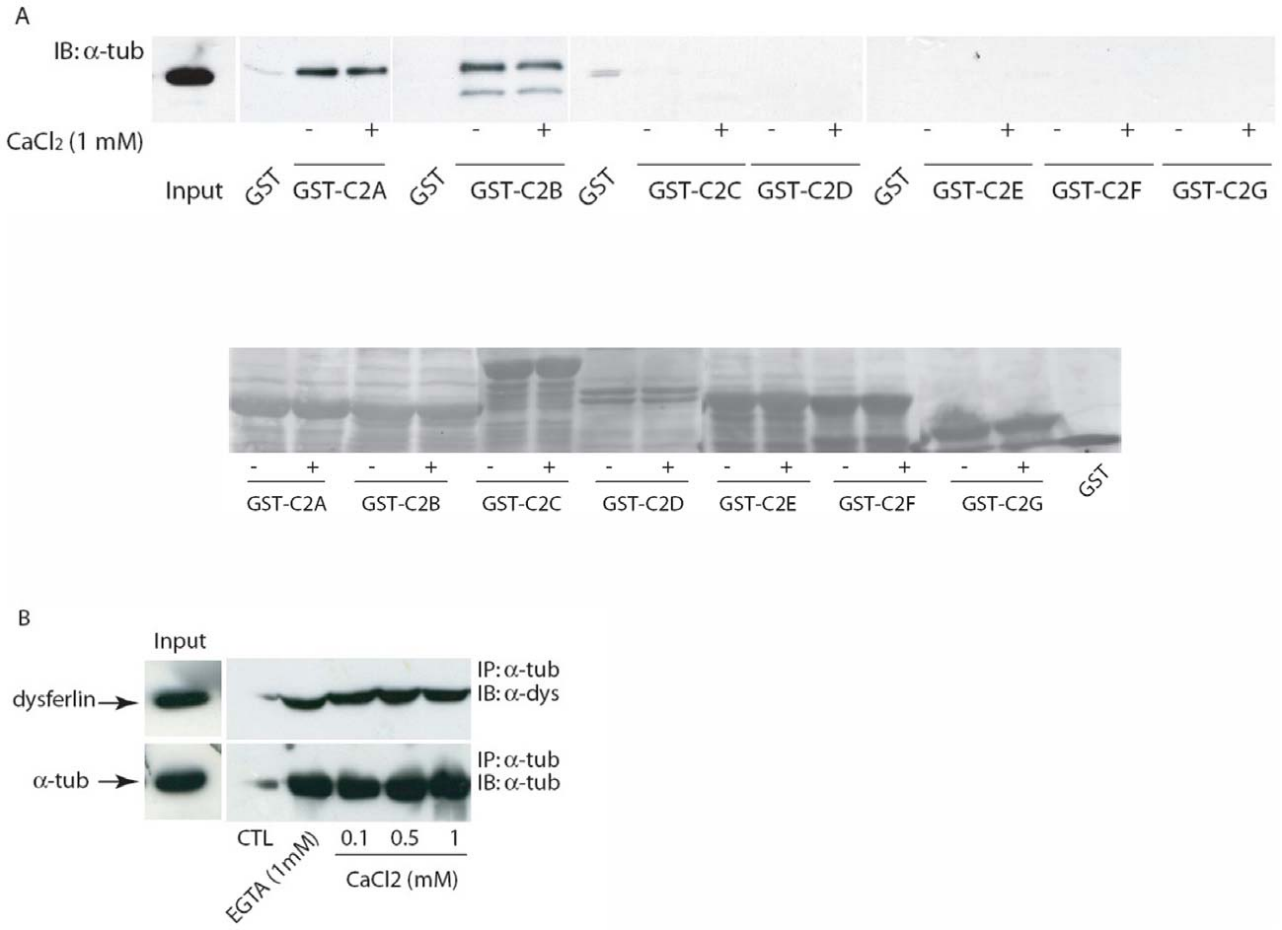
Alpha-tubulin interacts with dysferlin in a calcium-independent manner. **A.** Upper panel: Myoblast cell extracts were incubated with GST alone or the various GST-dysferlin C2 domain fusion proteins precoupled to glutathione-Sepharose 4B beads in the absence (−) or presence (+) of 1 mM calcium. The bound proteins were separated on SDS-PAGE followed by Western blot analysis using anti-alpha-tubulin antibody. Lower panel: nitrocellulose membrane of GST-dysferlin C2 domains with adsorbed proteins from the cell extract stained with ponceau red. **B.** Co-immunoprecipitation of alpha-tubulin and dysferlin with anti-alpha-tubulin antibody from mouse skeletal muscle homogenate in the presence of increasing calcium concentrations. Proteins were separated and detected with anti-alpha-tubulin and anti-dysferlin antibodies. As a control (CTL), protein A-Sepharose beads were incubated with muscle homogenate in the absence of anti-alpha-tubulin antibody.

Dysferlin C2 domains contain acidic residues in their Ca^2+^-binding loops, suggesting that their interactions with lipids and proteins could be modified by cations. We showed previously that the C2A domain of dysferlin is the only Ca^2+^-dependent lipid binding C2 domain that might be important for calcium-dependent membrane interactions in muscle [22]. We therefore decided to investigate the effect of Ca^2+^ on the interaction between dysferlin C2 domains and alpha-tubulin. The interaction with the C2A and C2B domains of dysferlin both demonstrated calcium-independent binding activity. No binding was found with the other dysferlin C2 domains in either the presence or absence of Ca^2+^ (Fig. 5A). Since the conformation of these individual domains could be altered under physiological conditions, we evaluated the possibility that these binding interactions could be different in muscle expressing full-length dysferlin. We performed a Ca^2+^ titration experiment for the binding interaction of alpha-tubulin and dysferlin in mouse muscle tissue. Analysis of the co-immunoprecipitates revealed that Ca^2+^ was not required for alpha-tubulin binding by native dysferlin in mouse muscle tissue (Fig. 5B).

### Dysferlin interacts with microtubules

**Figure 6:**
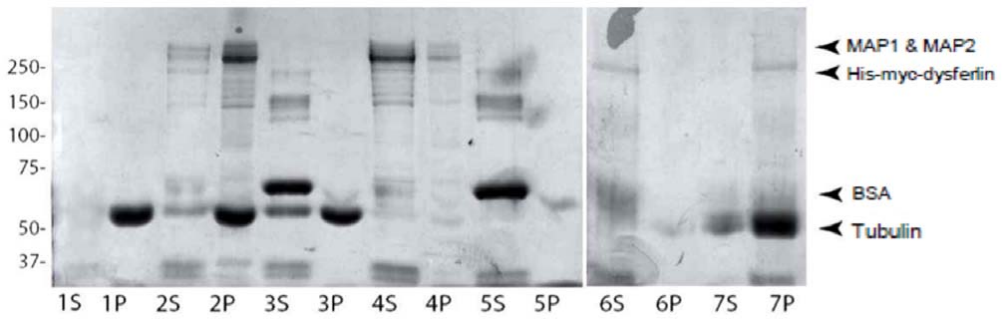
Dysferlin binds to microtubules. His-myc-dysferlin purified on Ni-NTA beads was incubated with polymerized microtubules. Reactions were resolved by SDS-PAGE stained with SimplyBlue SafeStain. Arrows point to His-myc-dysferlin, tubulin, BSA, and to MAP1&MAP2 of the microtubule-associated protein fraction (MAPF), which includes MAP2A, MAP2B, MAP1 and tau. S: Soluble phase, P: Pellet. Lane 1: Microtubules alone, lane 2: Microtubules incubated with MAPF, lane 3: Microtubules incubated with BSA, lane 4: MAPF alone, lane 5: BSA alone, lane 6: Purified His-myc-dysferlin alone, lane 7: Microtubules incubated with purified His-myc-dysferlin.

Alpha-tubulin exists in cells as soluble monomers or as soluble heterodimers together with beta-tubulin. The tubulin heterodimers serve as building blocks for the growing polymers of microtubules in cells. Hence, we examined whether dysferlin could bind to microtubules by using an *in vitro* microtubule sedimentation assay. Recombinant His-myc-dysferlin was incubated with polymerized microtubules. BSA was used as a negative control and MAPF (microtuble binding fraction) containing MAP2A, MAP2B, MAP1 and tau as a positive control, where MAP2 constitutes 70% of MAPF. His-myc-dysferlin and MAPF proteins were found to pellet with microtubules, whereas BSA did not (Fig. 6). In addition, both dysferlin and MAPF did not pellet in the absence of microtubules and remained in the soluble fraction.

**Figure 7:**
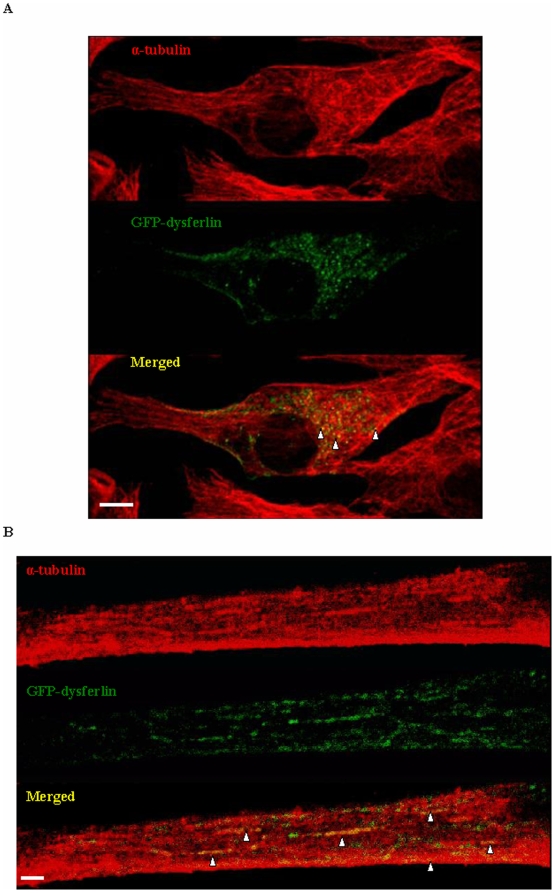
Alpha-tubulin co-localizes with dysferlin. GFP-dysferlin was expressed in C2C12 myoblasts and myotubes. The localization of GFP-dysferlin was compared to that of endogenous alpha-tubulin stained with anti-alpha-tubulin antibody by confocal microscopy. The co-localization of dysferlin with alpha-tubulin is revealed in the merged image. Arrowheads point to areas of co-localization. *Scale bar* represents 10 µm.

To further confirm this molecular interaction, we examined the cellular distribution of dysferlin and microtubules in C2C12 myoblasts and myotubes. We transiently transfected C2C12 myoblasts with GFP-dysferlin and induced myotube formation. Cells were fixed and stained for alpha-tubulin (Fig. 7). The staining pattern of alpha-tubulin differed in myoblasts from the pattern seen in myotubes. In the former, we observed the typical mesh-like distribution of microtubules in the cytoplasm, which originates from a microtubule organizing center near the nucleus. In myotubes, this pattern is changed and adopts a longitudinal configuration along the myotube axis. In myoblasts, GFP-dysferlin was found to partially co-localize with alpha-tubulin in the perinuclear region, as well as in vesicular structures (Fig. 7A). In myotubes, GFP-dysferlin partially co-localized with alpha-tubulin in thin longitudinal structures indicative of microtubules (Fig. 7B).

## Discussion

Dysferlin is a multi-domain transmembrane protein implicated in skeletal muscle surface membrane repair. The function of dysferlin in membrane repair is supported by several lines of evidence obtained through the study of dysferlinopathic animal models [12], [13], [26], [27], as well as through the molecular characterization of dysferlin's protein and lipid binding partners [6], [7], [8], [9], [12], [13], [22]. Several muscle proteins were shown to interact with dysferlin, such as MG53, affixin, annexins A1 and A2, caveolin-3, calpain-3, the dihydropyridine receptor and AHNAK in mouse or human skeletal muscle [6], [8], [9], [10], [12], [28]. Some of these proteins were also shown to be implicated in the repair of membrane tears of muscle fibres like annexins which are fusogenic proteins with affinity for membrane lipids and phosphoinositides [14], [29] or MG53, a protein implicated in vesicular trafficking [12]. Thus, the concept of a protein repair complex is emerging and probably involves several other proteins and lipid components. The role of dysferlin as a Ca^2+^ sensor is well accepted and similarly to otoferlin, a Ca^2+^ sensor of the inner ear hair cell ribbon synapse, may promote vesicle fusion to the plasma membrane in skeletal muscle fibres [30]. The discovery of other dysferlin molecular interactors will undoubtedly be useful in the understanding of dysferlin biology.

In this study, we undertook a proteomic search for additional dysferlin protein binding partners through the analysis of immunoprecipitated proteins from mouse skeletal muscle using Liquid chromatography-Mass spectrometry (LC-MS/MS). We identified alpha-tubulin as a novel dysferlin binding protein and showed that it interacts with dysferlin through the C2A and C2B domains in a calcium-independent manner. Moreover, we showed that dysferlin was able to bind to polymerized tubulin in form of microtubules *in vitro* and *in vivo*.

Microtubules are involved in maintenance of cell morphology, distribution and transport of membrane proteins, cell polarization and motility. Molecular details of these processes are still under investigation; however, a novel class of microtubule plus-end-binding proteins shed new light on transport between the endoplasmic reticulum (ER), Golgi apparatus and the plasma membrane. Experiments using mammalian epithelial cell lines have elucidated biosynthetic and recycling pathways for apical and basolateral plasma membrane proteins, and have identified components that guide apical and basolateral proteins along these pathways [31], [32]. Recent live-cell-imaging studies provide a real-time view of sorting processes in epithelial cells, including key roles for microtubules in protein transport to the plasma membrane [33], [34]. In this regard it is interesting that our study identified dysferlin as a microtubule binding protein, since dysferlin has to be transported to the surface plasma membrane of muscle cells.

We demonstrated that dysferlin co-localizes with microtubules in myoblasts as well as in myotubes, and we believe that this interaction is of significance despite the fact that the observed co-localization is only partial.

Another protein shown to interact, yet to only partially co-localize with microtubules is Annexin XI. Partial co-localization with microtubules was shown for Annexin XI in human epidermoid cancer cells (HEp-2) [35]. Annexins, like C2-domain proteins, are Ca^2+^ effector proteins that bind to negatively-charged phospholipids and provide a link between calcium signalling and membrane functions [36].

The trafficking of normal or mutant ferlin proteins in mammalian cells is under-characterized and only partial trafficking data is available. For example, endocytosed myoferlin is recycled back to the plasma membrane via the EHD2 protein, a carboxyl-terminal EH domain-containing protein implicated in surface membrane protein recycling [37]. An EHD2 mutant expressed in myoblasts led to the sequestration of myoferlin in recycling endosomes and inhibition of myoblast fusion [37]. Caveolin-3 maintains a plasma membrane pool of dysferlin by inhibiting dysferlin endocytosis through a clathrin-independent pathway [38]. The plasma membrane localization of dysferlin implies that it is translocated and inserted into the ER membrane after its synthesis in the cytosol and then targeted to the plasma membrane by the secretory pathway. Dysferlin localization remains a subject of argument. Full-length dysferlin was shown to localize to the T-tubule system and to translocate to the injury site in C2C12 myotubes [6], whereas other studies showed that dysferlin is localized to vesicles near the Golgi apparatus [21]. The trans-Golgi sorting mechanism and the type of vesicles used by dysferlin to reach the plasma membrane are not known.

Recently, MG53 was shown to interact with dysferlin and caveolin-3 to facilitate vesicle trafficking to the site of membrane injury and to mediate the movement of dysferlin to the site of injury [12].

It was previously shown that the C2A domain of dysferlin bound phosphoinositides and phosphatidylserine in a Ca^2+^-dependent fashion [22]. Dysferlin's C2A domain shares structural homology with the C2A domain of synaptotagmin I, which also binds similar phospholipids [39], [40]. Synaptotagmins play an essential role in neurotransmitter release by acting as a calcium sensor mediating calcium-dependent synaptic vesicle exocytosis [41], [42]. It was shown that tubulin interacts directly with both the C2A and C2B domains of synaptotagmin I [43]. For synaptotagmin I, it was suggested that direct tubulin binding provides a mechanism for retaining synaptic vesicles on microtubules as a readily releasable pool. Synaptic vesicles in the nerve terminal are stored in two distinct pools: the readily releasable pool, which is kept close to the active zone, and the reserve pool, which is stored at a distance from the plasma membrane [44], [45]. In another paradigm, in experiments performed in PtK2 (rat kangaroo kidney) cells, it has been shown that following membrane disruption, microtubule rearrangement occurs towards the site of injury [46]. Unifying these and our observations, we hypothesize that dysferlin is stored in a ready pool close to the cell surface through its interaction with microtubules. This interaction could be essential to ensure a ready pool of dysferlin required for membrane resealing.

We demonstrate that dysferlin's interaction with alpha-tubulin is mediated by the C2A and C2B domains in a calcium-independent manner. This is at odds with the described calcium-dependent phospholipid binding of dysferlin's C2A domain [22] and with the described calcium-dependent binding of synaptotagmin I to microtubules [43]. However, synaptotagmin IX was recently reported to bind alpha- and beta-tubulin in the absence of calcium in rat basophilic leukemia mast cells (RBL) [47], which is in line with our findings. Binding of synaptotagmin IX to beta-tubulin, but not to alpha-tubulin, was enhanced only at very high, non-physiological concentrations of Ca^2+^ in that study [47].

In conclusion, we have identified alpha-tubulin as a novel protein binding partner of dysferlin. Our findings demonstrate that dysferlin binds to alpha-tubulin through its C2A and C2B domains as well as to microtubules. It will be of great interest to determine the implications of this protein interaction on the plasma membrane homeostasis and on dysferlin trafficking in skeletal muscle.
